# Differential Safety and Lipid Control Efficacy of β‐1,3/1,6‐Glucan Oligosaccharides and Polysaccharides Derived From *Ophiocordyceps dipterigena* BCC 2073 in Healthy Volunteers

**DOI:** 10.1002/fsn3.71379

**Published:** 2026-03-02

**Authors:** Numphung Rungraung, Niramol Muangpracha, Pakkapong Phucharoenrak, Wai Prathumpai, Dunyaporn Trachootham

**Affiliations:** ^1^ Institute of Nutrition Mahidol University Nakhon Pathom Thailand; ^2^ Biocontrol Technology Research Team, Integrative Crop Biotechnology and Management Research Group National Center for Genetic Engineering and Biotechnology, National Science and Technology Development Agency Pathum Thani Thailand

**Keywords:** β‐glucan, dietary supplement, fungi, oligosaccharides, polysaccharides, randomized control trial, safety

## Abstract

β‐1,3/1,6‐glucan is an immune‐modulating functional ingredient. To enhance solubility, β‐1,3/1,6‐glucan oligosaccharides were developed from polysaccharides through gamma‐irradiation. Nevertheless, whether their safety and efficacy profiles are different remains unclear. Our previous study identified 2000 mg/day as the maximum short‐term tolerable dose of *Ophiocordyceps dipterigena* BCC2073‐derived β‐1,3/1,6‐glucan oligosaccharides and polysaccharides. However, the long‐term safety of this dosage was unknown. This randomized, blinded, placebo‐controlled trial was conducted to evaluate their safety over 12 weeks. Ninety‐six healthy participants were randomly assigned to receive 2000 mg daily of either β‐glucans oligosaccharides, β‐glucans polysaccharides, or placebo capsules (*n* = 32 each group). Adverse symptoms, changes in body weight, defecation, hematological, and biochemical parameters, vital signs, and heart function were assessed using subject diaries, interviews, blood and urine tests, and electrocardiograms. No serious adverse events or changes in body weight, liver or renal function, complete blood counts, blood glucose levels, urinalysis, or electrocardiogram were observed in any of the groups. Notably, supplementation with β‐glucan oligosaccharides, but not polysaccharides, resulted in significant reductions in total cholesterol and LDL levels compared to the control group. Only the polysaccharides group had significant positive (easier defecation) and negative (constipation, loose stools) defecation‐related symptoms. The negative symptoms (found in 3%–19% of participants) were mild. These findings indicate that daily supplementation with 2000 mg of β‐1,3/1,6‐glucan oligosaccharides or polysaccharides for 12 weeks is safe in healthy individuals. The oligosaccharides demonstrated superior lipid‐lowering efficacy with fewer adverse events compared to the polysaccharides. Possible defecation‐related side effects of β‐1,3/1,6‐glucan polysaccharides should be considered.

**Trial Registration:** Thai Clinical Trial Registry: TCTR20240622005

## Graphical abstract

This randomized, blinded, placebo‐controlled trial uncovers the novel findings that daily supplementation with 2000 mg of β‐1,3/1,6‐glucan oligosaccharides or polysaccharides for 12 weeks is safe in healthy individuals. The oligosaccharides demonstrated superior lipid‐lowering efficacy with fewer adverse events than the polysaccharides. Possible defecation‐related side effects should be a concern for β‐1,3/1,6‐glucan polysaccharides.

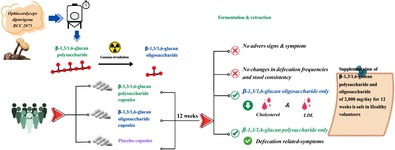

## 1 Introduction

β‐1,3/1,6‐glucans are naturally occurring polysaccharides found in various types of fungi, including yeast, mushrooms, and mold (Xu et al. 2024; Du et al. 2019). These glucans consist of a β‐1,3‐glucans backbone and β‐1,6‐glycosidic bonds branching (Xu et al. 2024; Du et al. 2019). Both β‐(1,3)‐glucan and β‐(1,6)‐structure contribute to the immune‐modulating effects of beta‐glucans (Han et al. 2020; Noss et al. 2013). To enhance solubility and biological activity, β‐glucan oligosaccharides have been developed from β‐glucan polysaccharides via either hydrolysis or gamma irradiation (Byun et al. 2008; Lei et al. 2015). Nevertheless, it remains unclear whether the safety of β‐glucan oligosaccharides differs from that of polysaccharides.

Currently, β‐1,3/1,6‐glucans derived from yeast are widely used in various applications. However, their use may be limited in individuals with yeast or chitin allergies. Additionally, the purity of the glucans can be compromised by gluten contamination, particularly when using by‐products from Baker's yeast in the bakery industry (Thomas et al. 2022). As an alternative, β‐1,3/1,6‐glucans from the fungus *Ophiocordyceps dipterigena* BCC 2073 have recently been developed (Methacanon et al. 2005). These β‐glucans are potent inducers of IL‐8, a cytokine that promotes wound healing (Methacanon et al. 2005), and possess prebiotic properties (Prathumpai et al. 2015, 2019). To scale up the production of these β‐1,3/1,6‐glucans at an industrial level, a novel fermentation technique using specially formulated low‐cost media was employed, yielding β‐glucan polysaccharide with a molecular weight of 800–900 kDa (Prathumpai et al. 2017). These polysaccharides were then subjected to gamma irradiation to produce β‐glucan oligosaccharides with a molecular weight of 5–8 kDa, which exhibited superior IL‐8 stimulatory activity compared to their higher molecular weight counterparts (Methacanon et al. 2011).

Previous in vitro studies reported that shortening yeast β‐1,3/1,6‐glucans through gamma irradiation enhanced their solubility, reduced viscosity, increased fat binding capacity and emulsifying properties, and boosted antioxidant and antibacterial activities (Khan et al. 2016). Additionally, an in vivo study in mice demonstrated that low‐molecular‐weight yeast beta‐glucan is more effective than its polysaccharide counterpart in promoting antioxidant activity, suppressing lipid peroxidation, enhancing lymphocyte proliferation, and regulating cytokine (IL‐2, IFN‐gamma) production (Lei et al. 2015). However, the clinical safety and lipid‐lowering effects of β‐glucan oligosaccharides compared to polysaccharides remain unclear. Our previous human study showed that 2000 mg/day is the maximum short‐term tolerable dose of β‐1,3/1,6‐glucan oligosaccharides and polysaccharides derived from *O. dipterigena* BCC2073 (Muangpracha et al. 2025). However, the safety of this dose for long‐term consumption remained unclear. Therefore, this randomized, double‐blinded, placebo‐controlled study was conducted to evaluate the safety of β‐glucan oligosaccharides and polysaccharides over a 12‐week period.

## 2 Materials and Methods

### 2.1 Ethical Aspects and Setting

The study protocol (MU‐CIRB 2023/198.1906) was approved by the Mahidol University Central Institutional Review Board (MU‐CIRB), approval number COA No. MU‐CIRB2023/113.2407. This research was conducted in accordance with the International Council for Harmonization of Technical Requirements for Pharmaceuticals for Human Use Good Clinical Practice (ICH‐GCP) guideline and the Declaration of Helsinki. Informed written consent was obtained from all participants before enrollment. The study protocol, titled “Safety for Continuous Intake of fungal Beta‐Glucan polysaccharide and Beta‐Glucan oligosaccharide in healthy volunteers” was registered in the Thai Clinical Trial Registry and is available at https://www.thaiclinicaltrials.org/#. The registration number is TCTR20240622005.

### 2.2 Study Design, Blinding, Random Allocation, and Concealment

A randomized, blinded, placebo‐controlled trial design was employed. Participants who passed the screening process were randomly assigned to one of three groups: two intervention groups, which received either β‐glucan polysaccharides or β‐glucan oligosaccharides, and a control group that received placebo capsules. All participants were provided with unlabeled sachets containing their assigned product to ensure blinding. In addition, both the laboratory personnel conducting the analyzes and the statistician performing the data evaluation were blinded to the group assignments.

### 2.3 Participants

The inclusion criteria for participant screening were as follows: healthy individuals aged 20–55 years with no systemic diseases and a body mass index (BMI) ≤ 30 kg/m^2^. Eligible participants had blood tests result within the past 6 months that fell within normal acceptable ranges: complete blood count (CBC) with hemoglobin level of 11–16 g/dL for females and 12–18 g/dL for males, and white blood cell count (WBC) of 4000–10,000 cells/mm^3^; fasting plasma glucose (FPG) ≤ 125 mg/dL and HbA1c ≤ 5.8%; total cholesterol to HDL ratio ≤ 5:1; alanine aminotransferase (ALT) ≤ 60 U/L; and estimated glomerular filtration rate (eGFR) ≥ 90 mL/min/1.73 m^2^. Additionally, participants were required to present with normal vital signs, including a blood pressure of ≤ 160/90 mmHg and a resting heart rate of ≤ 100 beats per minute.

Exclusion criteria included: a history of exposure to individuals infected with COVID‐19, known allergy to Cordyceps or other herbal extracts; inability to attend follow‐up visits every 4 weeks, pregnancy or plan to become pregnant within the next 3 months; breastfeeding; regular use of medications, herbal products or dietary supplements; alcohol consumption exceeding 14 drinks per week for male or 7 drinks per week for females or inability to abstain from alcohol during the study period; smoking more than 10 cigarettes per day; coagulation disorders (e.g., idiopathic thrombocytopenic purpura); and medical conditions deemed unsuitable for β‐Glucan supplementation, such as severe gastritis or irritable bowel syndrome. Written informed consent was obtained from all participants before data collection.

### 2.4 Sample Size and Power

The sample size for this study was calculated using G*Power version 3.1.9.4 (Heinrich‐Heine‐Universität Düsseldorf, Düsseldorf, Germany) (Kang 2021). As the study aimed to compare means across three groups, a large theoretical effect size (*f* = 0.4) was applied for one‐way ANOVA (Kim 2016). With a statistical power of 0.80 and a significance level of 0.05, the required sample size was determined to be 28 participants per group. To account for an anticipated dropout rate of 15%, the sample size was increased to 32 participants per group, resulting in a total of 96 participants.

### 2.5 Intervention and Materials

Our previous study demonstrated that daily consumption of 2000 mg of β‐glucan polysaccharides and β‐glucan oligosaccharides was safe for 2‐week periods (Muangpracha et al. 2025). Therefore, the present study aimed to evaluate the long‐term safety of this dosage. Participants were instructed to take 2000 mg per day (four capsules of 500 mg each) of either β‐glucan oligosaccharides, β‐glucan polysaccharides, or placebo, approximately 30 min before breakfast, once daily, for a duration of 12 weeks.

Both β‐glucan and placebo capsules were manufactured by Asia Star Trade Co. Ltd. (Bangkok, Thailand) in a facility certified to Good Manufacturing Practice (GMP) standards. The β‐glucan polysaccharides was produced through the fermentation of *Ophiocordyceps dipterigena* BCC 2073 using a specially optimized medium, as previously described (Prathumpai et al. 2017). The molecular weight of 500–800 kDa reported in published studies on the characterization of β‐glucans was from laboratory‐scale production. However, the actual products used in this study were produced on an industrial scale with a molecular weight of around 900 kDa. To obtain β‐glucans oligosaccharides, the polysaccharides was subjected to 100 kGy gamma irradiation using a cobalt‐60 irradiator, resulting in a product with a molecular weight of approximately 5–8 kDa (Methacanon et al. 2011). The β‐glucans were then drum‐dried, milled into a powder, and encapsulated into size 0 capsules at the specified dosage. The purity of the β‐glucan product was 80%–85%. The capsule only contains the powder of β‐glucan oligosaccharides or polysaccharides with no excipient, adhesive, or binder. Therefore, the purity of β‐glucan is determined by the amount of β‐glucan divided by the total weight. First, the amount of total glucan (α‐glucan and β‐glucan) and α‐glucan was determined. Second, the amount of β‐glucan is obtained by subtracting the α‐glucan content from the total glucan value. Third, the amount of β‐glucan was divided by the total weight to calculate the purity. A detailed method has been described in the Supplemental data.

The actual study samples are capsules of β‐glucan oligosaccharides or polysaccharides, with no other components inside the capsule. The purity of 82.5% is the amount of β‐glucan per 100 g of product. The fungal beta‐glucan is a (1➔3)‐β‐D‐glucan backbone, substituted at O‐6 with side chains of (1➔6)‐β‐D‐pyranosyl units, and contains 1.86% arabinose, 29.08% mannose, 25.86% galactose, and 43.05% glucose. As analyzed by size exclusion chromatography, the actual study samples had size distributions of 900 kDa for β‐glucan polysaccharides and 5 kDa for oligosaccharides (Figure S1).

Placebo capsules were identical in size, shape, and color, and consisted of empty size 0 capsules made from bovine gelatin and 0.7778% titanium dioxide. All products met the microbiological and chemical safety requirements in accordance with the Thai Ministry of Public Health Notifications No. 293, 309, 405, 411, 414, and 416 (Notification of the Ministry of Public Health (No. 293) B.E. 2548 2005; Notification of the Ministry of Public Health (No. 309) B.E. 2550 2007; Notification of the Ministry of Public Health (No. 405) B.E. 2562 Issued by the Food Act B.E. 2522 2019; Notification of the Ministry of Public Health (NO. 411), B.E. 2562 (2019) Issued Under the Food Act, B.E. 2522 (1979) 2019; Notification of the Ministry of Public Health (No. 414) B.E. 2563 2020 and Notification of the Ministry of Public Health (No. 416) B.E. 2563 2020). All capsules used in the study were derived from the identical manufacturing batches.

### 2.6 Study Procedure

This study was conducted at the Institute of Nutrition, Mahidol University. Participants who passed the screening were randomly assigned to one of three groups. Each participant consumed the assigned product once daily, approximately 30 min before breakfast, for 12 consecutive weeks. The daily dose consisted of one sachet containing 2000 mg (four capsules of 500 mg each). Participants documented their daily intake in subject diaries and recorded their dietary intake 3 days per week (two weekdays and one weekend day) to capture overall dietary patterns. These records were reviewed by the investigator at each follow‐up visit to ensure adherence to the protocol. Participants were also instructed to maintain their usual level of physical activity throughout the study. Follow‐up visits were scheduled at weeks 4, 8, and 12 during the intervention period. After the 12‐week supplementation phase, participants discontinued product consumption and returned for a final follow‐up at week 14 (2 weeks post‐intervention). Safety assessments, including blood tests, urinalysis, body weight, vital signs, and electrocardiograms (EKGs), were conducted at baseline (week 0) and at weeks 4, 8, 12, and 14. Adverse symptoms were monitored daily through participant self‐reports in subject diaries and confirmed by researcher interviews during each follow‐up visit. The study's conceptual framework is presented in Figure S2.

### 2.7 Outcome Measurement

The safety and potential efficacy of β‐glucan capsules (comprising β‐glucan polysaccharide and β‐glucan oligosaccharide) were assessed in comparison to a placebo using several clinical parameters. These included body weight, vital signs (blood pressure and pulse rate), and laboratory tests. Clinical laboratory assessments included FPG; liver function tests [aspartate aminotransferase (AST), ALT, and total bilirubin]; renal function markers [blood urea nitrogen (BUN) and creatinine (CR)]; lipid profile [total cholesterol, high‐density lipoprotein (HDL), low‐density lipoprotein (LDL), and total triglycerides]; CBC; urinalysis; and EKGs. Adverse symptoms were monitored throughout the study using participant‐maintained subject diaries and follow‐up interviews conducted by researchers at each scheduled visit. Body weight was measured using a bioelectrical impedance analyzer (TANITA BC‐730, Tanita Corporation, Tokyo, Japan), while blood pressure and pulse rate were assessed with a digital blood pressure monitor (OMRON HEM‐7156A).

### 2.8 Statistical Analysis

Numerical data were summarized as mean ± standard deviation (SD), while categorical data were presented as frequencies and percentages. Dietary intake data from food records were analyzed using INMUCAL‐Nutrient Version 4.0. Graphs and statistical analyzes were performed using GraphPad Prism Version 10.4.2. Baseline characteristics between the intervention and control groups were compared using Fisher's exact test for categorical variables and unpaired *t*‐tests for continuous variables. Changes within each group over time were assessed using the Kruskal–Wallis test. Differences in changes over time between groups were analyzed using mixed‐effects analysis followed by Sidak's multiple comparisons test. A *p*‐value of < 0.05 was considered statistically significant.

## 3 Results

### 3.1 Participant Flow Chart

**FIGURE 1:**
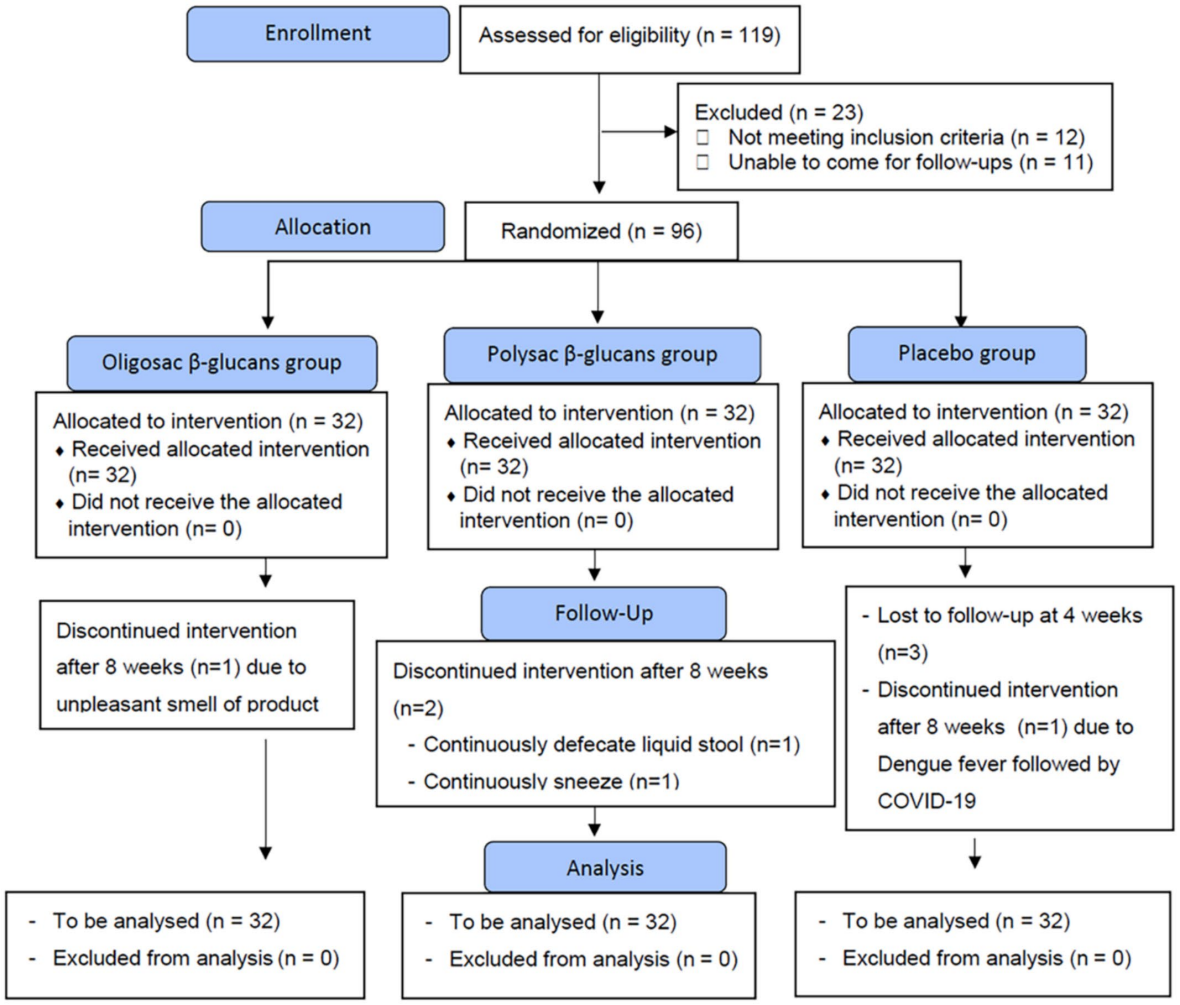
CONSORT participants' flowchart.

This study was conducted from October 2023 to February 2024. Figure 1 presents the participant flow diagram in accordance with the Consolidated Standards of Reporting Trials (CONSORT) guidelines. A total of 119 individuals were initially screened for eligibility, of whom 96 met the inclusion criteria and were randomly assigned into three groups: β‐glucan oligosaccharides (*n* = 32), β‐glucan polysaccharides (*n* = 32), and placebo (*n* = 32). During the study, one participant from the β‐glucan oligosaccharides group, two from the β‐glucan polysaccharides group, and three from the placebo group withdrew before the study was completed. As the majority of these participants had completed at least 8 weeks of the intervention, all available data were included in the intention‐to‐treat (ITT) analysis. Statistical comparisons were conducted using mixed‐effects models followed by Tukey's multiple comparison tests.

### 3.2 Baseline Demographic, Clinical Chemistry, and Hematology Characteristics of the Participants

No significant differences were observed among the three groups in terms of gender, age, BMI, presence of congenital diseases, smoking status, alcohol consumption, systolic and diastolic blood pressure, or pulse rate (*p* > 0.05; Table S1). Similarly, there were no significant differences in baseline hematological and biochemical parameters, including liver function, renal function, FPG, lipid profiles, or CBCs across the groups (*p* > 0.05; Table 1).

**TABLE 1:** Baseline hematological and biochemical blood parameters of all participants.

Parameter	β‐glucans oligosaccharide Group (*n* = 32)	β‐glucans polysaccharide Group (*n* = 32)	Control Group (*n* = 32)	*p*
WBC (cells/mm^3^)	6.12 ± 1.36	6.49 ± 1.35	6.58 ± 2.01	0.338
RBC (million cells/mm^3^)	4.72 ± 0.46	4.54 ± 0.49	4.64 ± 0.50	0.468
Hemoglobin (g/dL)	12.79 ± 1.20	12.67 ± 1.13	12.90 ± 1.47	0.741
Hematocrit value (%)	39.00 ± 3.27	38.38 ± 3.05	39.16 ± 3.68	0.891
MCV (fL)	82.21 ± 7.79	83.96 ± 7.09	83.65 ± 7.15	0.429
MCH (pg)	27.08 ± 3.08	27.22 ± 3.53	27.86 ± 2.75	0.444
MCHC (g/dL)	32.89 ± 1.21	33.12 ± 0.97	32.96 ± 1.16	0.944
RDW (%)	13.80 ± 1.90	13.42 ± 0.89	13.53 ± 1.39	0.944
Platelet (×10^3^ cells/mm^3^)	297.50 ± 65.27	299.25 ± 78.78	279.06 ± 74.57	0.186
Neutrophils (%)	53.78 ± 8.43	55.81 ± 7.88	53.97 ± 9.08	0.561
Lymphocytes (%)	35.09 ± 7.57	32.75 ± 7.03	34.41 ± 8.41	0.785
Monocytes (%)	6.69 ± 2.82	7.25 ± 1.52	7.13 ± 2.52	0.82
Eosinophil (%)	3.09 ± 2.57	3.38 ± 2.55	3.59 ± 3.19	0.791
Basophil (%)	0.66 ± 0.60	0.81 ± 0.47	0.84 ± 0.51	0.27
Fasting plasma glucose (mg/dL)	93.75 ± 6.12	95.69 ± 6.07	93.63 ± 6.4	0.32
HbA1C (%)	5.42 ± 0.36	5.43 ± 0.26	5.44 ± 0.25	0.943
Total cholesterol (mg/dL)	207.20 ± 29.79	205.90 ± 31.94	205.30 ± 27.50	0.923
Triglyceride (mg/dL)	108.40 ± 35.86	98.03 ± 31.75	104.10 ± 27.54	0.241
HDL cholesterol (mg/dL)	65.06 ± 11.83	66.91 ± 12.89	65.53 ± 12.20	0.906
LDL cholesterol (mg/dL)	120.50 ± 28.47	119.50 ± 26.69	119.00 ± 25.28	0.977
Total cholesterol: HDL ratio	3.27 ± 0.69	3.16 ± 0.65	3.22 ± 0.64	0.701
BUN (mg/dL)	10.3 ± 1.89	10.88 ± 2.51	11.00 ± 3.23	0.644
Creatinine (mg/dL)	0.72 ± 0.11	0.72 ± 0.11	0.70 ± 0.0	0.671
eGFR (mL/min/1.73m^2^)	110.4 ± 10.37	110.7 ± 11.39	110 ± 11.52	0.944
AST (U/L)	19.5 ± 5.89	17.56 ± 8.67	19.00 ± 5.58	0.706
ALT (U/L)	16.53 ± 5.58	15.06 ± 5.07	16.25 ± 6.84	0.356
Total bilirubin (mg/dL)	0.45 ± 0.10	0.43 ± 0.05	0.44 ± 0.09	0.970
Serum sodium (mEq/L)	137.90 ± 1.900	137.40 ± 1.85	138.30 ± 1.41	0.096
Serum potassium (mEq/L)	4.21 ± 0.35	4.08 ± 0.29	4.19 ± 0.51	0.295
Serum chloride (mEq/L)	101.40 ± 1.68	101.50 ± 1.59	101.80 ± 1.74	0.676
Serum carbon dioxide (mEq/L)	21.16 ± 1.65	20.66 ± 1.86	20.94 ± 2.49	0.429

*Note:* Data are shown as mean ± SD; *p*‐value from one‐way ANOVA for neutrophils, lymphocytes, and LDL cholesterol and Kruskal‐Wallis tests for other parameters.

Abbreviations: ALT, alanine aminotransferase; AST, aspartate transaminase; BUN, blood urea nitrogen; eGFR, estimated glomerular filtration rate; HBA1C, glycosylated hemoglobin; HDL, high‐density lipoprotein; LDL, low‐density lipoprotein; MCH, mean cell hemoglobin; MCHC, mean cell hemoglobin concentration; MCV, mean cell volume; RBC, red blood cell count; RDW, red blood cell distribution width; WBC, white blood cell count.

### 3.3 Compliance

Most participants followed the procedure as instructed. Compliance was monitored by counting the number of capsules in the returned package after the study was completed, as well as through self‐reporting from participants in the subject diary. As shown in Table S2, the adherence to capsule consumption in all groups is about 99%.

### 3.4 Adverse Symptoms

No adverse symptoms were reported in any of the groups (β‐glucans oligosaccharides, β‐glucans polysaccharides, or control) throughout the study period (Table S3).

### 3.5 Changes in Body Weight

The average body weight remained unchanged across all groups, with no significant differences observed between the groups (Figure S3A,B).

### 3.6 Changes in FPG and HbA1C

The average FPG was slightly reduced in all groups, with no significant differences between groups (Figure S4A). In contrast, the average HbA1c levels were significantly reduced in all groups after 12 weeks, with *p* < 0.001, *p* < 0.05, and *p* < 0.05 for the oligosaccharide, control, and polysaccharide groups, respectively (Figure S4B). Although the oligosaccharide group showed a trend toward a more pronounced decrease in HbA1c compared to the other groups, the inter‐group differences were not statistically significant (Figure S4C).

### 3.7 Changes in Blood Lipid Profile

**FIGURE 2:**
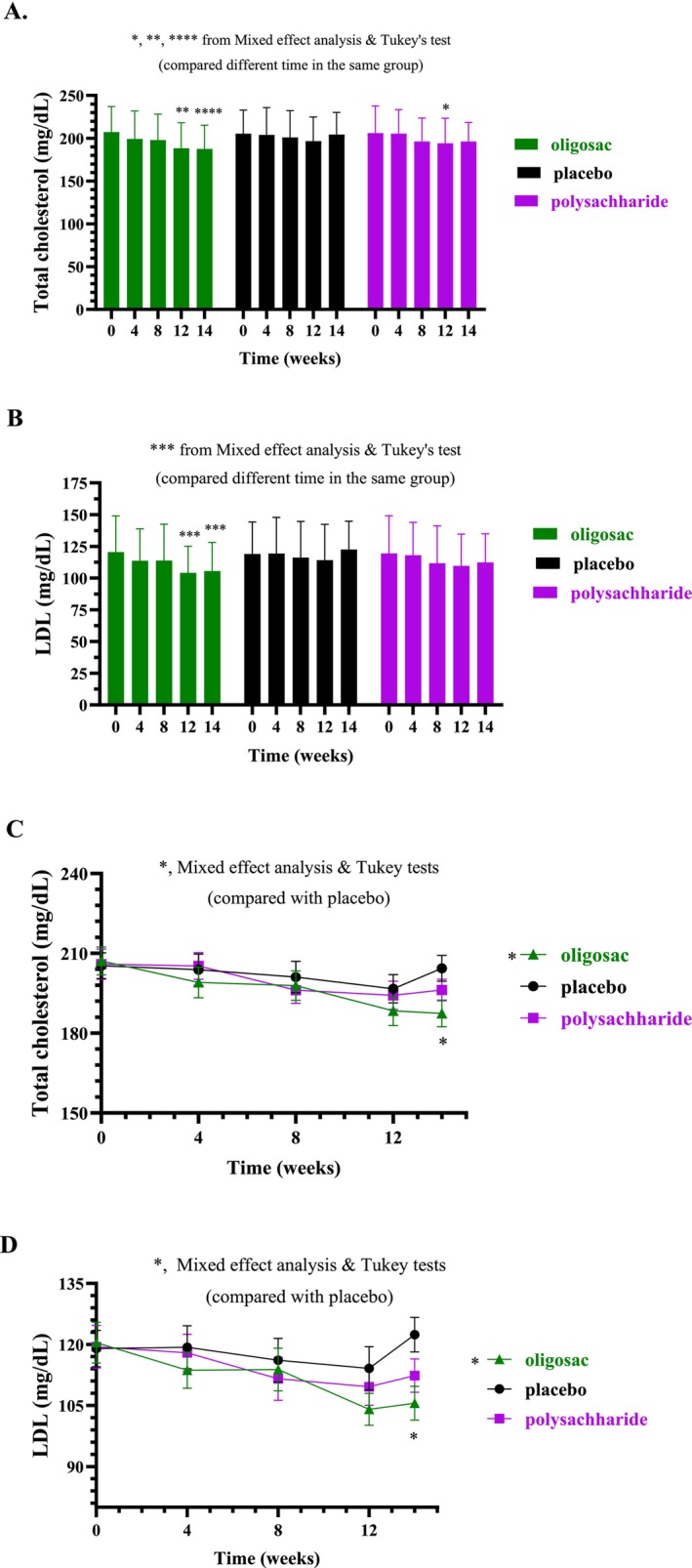
Changes in total cholesterol and LDL after intake of β‐glucans oligosaccharide (green), β‐glucans polysaccharide (purple), and control (black) groups supplementation for 4, 8, and 12 weeks and 2 weeks after stopping intake (14 weeks). The bar graphs show the mean and standard deviation of total cholesterol (A) and LDL (B) with *p*‐value analyzed by mixed effect analysis and Tukey's test (compare different time in the same group). The line graph shows the mean and standard deviation of the volunteer's total cholesterol (C) and LDL (D), with *p*‐value obtained from mixed effect analysis followed by Tukey's test. *, **, ***, and ****, mean *p* < 0.05, 0.01, 0.001, and 0.0001, respectively. LDL, low‐density lipoprotein.

As shown in Figure 2A, the average total cholesterol level tended to decrease after 4 weeks and 8 weeks of β‐glucans oligosaccharides and polysaccharides intakes, respectively. A significant reduction was observed after 12 weeks of intake for both β‐glucans oligosaccharides, and polysaccharides, compared to their respective baseline values (*p* < 0.01 and *p* < 0.05, respectively). However, 2 weeks after discontinuing the intervention, the average total cholesterol level remained significantly lower than baseline only in the oligosaccharides group (*p* < 0.0001), but not in the polysaccharides group. As shown in Figure 2B, a significant reduction in LDL levels compared to baseline values was observed only in the β‐glucan oligosaccharides group after 12 weeks of intake and persisted for 2 weeks post‐intervention (*p* < 0.001). Interestingly, when comparing the groups, Figure 2C,D consistently demonstrate that the total cholesterol and LDL levels in the oligosaccharides groups are significantly lower than those in the control groups (*p* < 0.05). As shown in Figure S5A,B, the average triglyceride and HDL levels remained unchanged across all groups, with no significant differences observed between them.

### 3.8 Changes in Kidney Function

**FIGURE 3:**
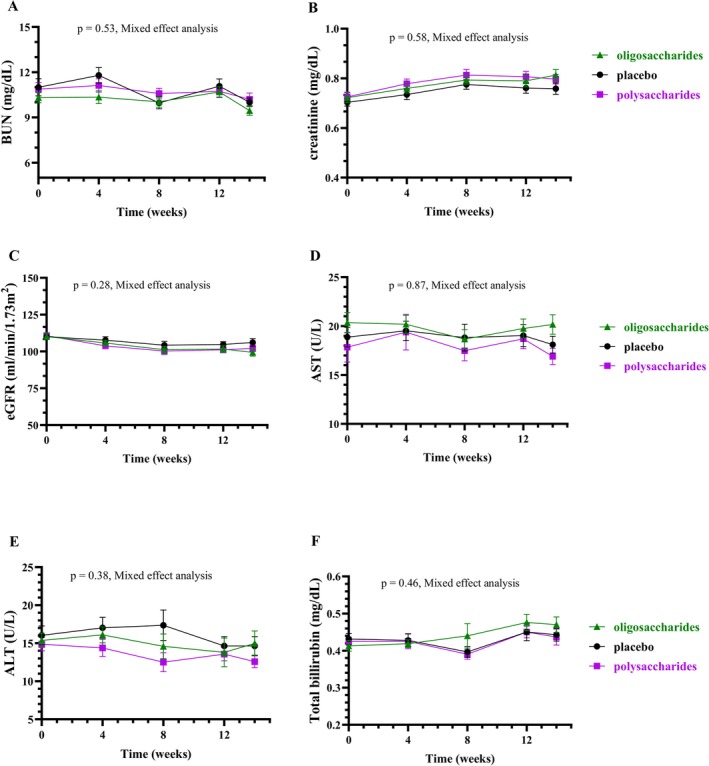
Changes in kidney and liver function. The line graph shows the mean and standard deviation of the volunteer's BUN (A), creatinine (B), eGFR (C), AST (D), ALT (E), and total bilirubin (F) after the β‐glucans oligosaccharide (green), β‐glucans polysaccharide (purple), and control (black) group supplementation for 4, 8, and 12 weeks and 2 weeks after stopping intake (14 weeks), with *p*‐value obtained from Mixed effect analyzes. Abbreviations: ALT, alanine aminotransferase; AST, aspartate transaminase; BUN, blood urea nitrogen; eGFR, estimated glomerular filtration rate.

As shown in Figure 3A–C, average BUN, creatinine, and eGFR levels remained stable across all groups, with no significant differences between them.

### 3.9 Changes in Liver Enzymes and Function

As shown in Figure 3D–F, average AST, ALT, and total bilirubin levels remained unchanged across all groups, with no significant inter‐group differences.

### 3.10 Changes in Hematological Parameters

As shown in Figure S6A–I, all hematological parameters—including WBC, RBC, platelets, Hb, Hct, RDW, MCV, MCH, and MCHC—remained stable across all groups, with no significant inter‐group differences.

### 3.11 Daily Record of Food Consumption

As shown in Table S4, the average intake of energy, carbohydrates, dietary fiber, sugar, protein, fat, and cholesterol remained unchanged in all groups, with no significant inter‐group differences.

### 3.12 Changes in Defecation

Our previous dose‐escalation study found changes in defecation patterns in some participants after 2 weeks of β‐glucan polysaccharides or oligosaccharides intake (Muangpracha et al. 2025). Therefore, in this 12‐week study, defecation data were collected through interviews and subject diaries. Based on the interviews, both positive and negative changes in defecation were reported significantly more often in the β‐glucan polysaccharides group than in the control group (Table 2). Although the oligosaccharides group also exhibited some incidence of positive and negative symptoms at specific time points, the differences were not statistically significant compared to the control group. Positive effects included easier defecation and improved bowel movement frequency for individuals who previously experienced constipation. Negative effects included constipation, loose stools, and increased frequency of bowel movements for individuals who previously had regular bowel movements. One participant in the β‐glucan polysaccharides group withdrew from the study due to persistent frequent bowel movements (2–3 times per day for several weeks). Although no medical treatment was needed, the frequency interfered with her daily activities.

**TABLE 2:** Percentage of participants reporting positive and negative changes in defecation‐related symptoms.

Intervention group	Times				p*
	Week 4	Week 8	Week 12	Week 14	
**Positive changes**					
β‐glucans oligosaccharide	35	23	21	7	0.0614
β‐glucans polysaccharide	22	26	27	17	**
Control	31	25	7	11	
**Negative changes**					
β‐glucans oligosaccharide	10	7	14	4	0.0701
β‐glucans polysaccharide	19	3	10	10	**
Control	3	4	0	0	

*Note:* Data are shown as the percentage of participants, *p‐values were obtained from the Chi‐square test (compared with placebo), ** represents *p* < 0.01.

Notably, the incidence of negative changes in the β‐glucan polysaccharides group ranged from 3% to 19%, remaining well below the 33% threshold. Therefore, the 2000 mg/day dosage is considered tolerable and not indicative of toxicity (Le Tourneau et al. 2009; Kurzrock et al. 2021). Additionally, subject diaries revealed no significant differences in defecation frequency between the β‐glucan oligosaccharides, polysaccharides, and control groups (Figure S7). Stool consistency, as measured by the Bristol Stool Scale, also showed no significant changes within or between groups (Figure S8).

### 3.13 Changes in Urinalysis

As shown in Table 3, most participants in all groups maintained normal urine parameters throughout the study. At certain time points, a few participants in each group had detectable levels of WBCs, RBCs, or protein in their urine. Cloudy urine was observed in 4, 2, and 1 participants in the β‐glucan oligosaccharides, polysaccharides, and control groups, respectively. However, their blood tests indicated normal renal function, and they reported reduced water intake the day before the sample was collected. Since the cloudy urine occurred sporadically and was not persistent, it is considered a random event rather than an abnormal finding. Participants with detectable RBCs in urine reported menstruation on the day of sample collection.

**TABLE 3:** Number of participants with abnormal urinalysis parameters.

Parameter	Normal range	β‐glucans oligosaccharide group (*n* = 32)					β‐glucans polysaccharide group (*n* = 32)					Control Group (*n* = 32)				
		Baseline	Week 4	Week 8	Week 12	Week 14	Baseline	Week 4	Week 8	Week 12	Week 14	Baseline	Week 4	Week 8	Week 12	Week 14
Color	Colorless/yellow	0	0	0	0	0	0	0	0	0	0	0	0	0	0	0
Appearance	Clear	0	4 (cloudy)	0	0	0	0	0	1	0	1 (turbid)	0	0	1 (cloudy)	0	1
Specific gravity	1.003–1.030	0	0	0	0	0	0	0	0	0	0	0	0	0	0	0
pH	5.0–8.0	0	0	0	0	0	0	0	0	0	0	0	0	0	0	0
Leukocyte	Negative	0	2 (1+, 3+)	0	0	0	0	0	0	0	1 (1+)	0	0	0	0	0
Nitrite	Negative	0	1 (1+)	0	0	0	0	0	0	0	0	0	0	0	0	0
Glucose	Negative	0	1 (trace)	0	0	0	0	0	0	0	0	0	0	0	0	0
Protein	Negative	0	1 (1+)	0	0	1 (1+)	0	0	1 (1+)	0	0	1 (1+)	0	0	1 (1+)	0
Bilirubin	Negative	0	0	0	0	0	0	0	0	0	0	0	0	0	0	0
Ketone	Negative	0	0	0	0	0	0	0	0	0	0	0	0	0	0	0
Urobilinogen	Normal	0	0	0	0	0	0	0	0	0	0	0	0	0	0	0
Blood	Negative	0	1 (1+)	0	0	0	0	0	0	1 (3+)	0	1 (1+)	1 (4+)	2 (2+)	0	0
WBC	0–1 cell/HP	0	2 (5–10, 10–20)	0	0	0	0	0	0	0	1 (5–10)	0	0	0	0	0
RBC	0–1 cell/HP	0	1 (5–10)	0	0	0	0	0	1 (1–2)	1 (30–50)	0	0	1 (50–100)	2 (20–30)	0	1 (2–3)
Squamous epithelial cell	0–1 cell/HP		2 (2–3, 3–5)		1 (1–2)	1 (1–2)	0	0	1 (3–5)	1 (3–5)	1 (2–3)	1 (3–5)	1 (3–5)	0	1 (5–10)	2 (1–2, 2–3)
Bacteria	Few	0	2 (3+, numerous)	0	0	0	0	0	0	0	0	1 (3+)	0	0	0	0

*Note:* Data are expressed as the number of participants with abnormal values, *n* (values).

Abbreviations: RBC, red blood cell; WBC, white blood cell.

### 3.14 Changes in EKG

As shown in Figure S9, there were no significant changes in EKG profiles across all groups and no significant inter‐group differences were observed. The proportion of participants with normal EKGs remained consistent throughout the study.

## 4 Discussion

Previous *in vitro* and *in vivo* studies have shown that β‐glucan oligosaccharides derived from yeast, mushrooms, or cereals, produced via hydrolysis or gamma‐irradiation, exhibit greater solubility and enhanced functional activities compared to their original polysaccharides (Byun et al. 2008; Lei et al. 2015; Khan et al. 2016; Zhong et al. 2015). However, their comparative safety profiles have not been well established. In this randomized, double‐blinded, placebo‐controlled trial, we demonstrate that daily supplementation with 2000 mg of β‐1,3/1,6‐Glucan oligosaccharides or polysaccharides from *Ophiocordyceps* *dipterigena* BCC 2073 for 12 weeks is safe in healthy volunteers. The oligosaccharides were associated with better lipid‐lowering efficacy and fewer adverse events. In contrast, defecation‐related side effects occurred in 3%–19% of participants in the polysaccharides group. To our knowledge, this is the first study to reveal differential safety and efficacy profiles between β‐Glucan oligosaccharides and polysaccharides, with the oligosaccharides showing a more favorable profile.

The European Food Safety Authority (EFSA) defines the acceptable daily intake (ADI) of yeast β‐1,3/1,6‐glucan polysaccharides as 375 mg/day for food supplements and 600 mg/day for foods for particular nutritional uses (PARNUTS) (EFSA Panel on Dietetic Products, Nutrition and Allergies (NDA) 2011). However, no safety limits have been established for β‐1,3/1,6‐glucan oligosaccharides, and β‐glucan from *Ophiocordyceps* *dipterigena* is considered a novel food. The results of this study could provide valuable evidence for establishing an ADI for fungal glucan oligosaccharides and polysaccharides. The ADI is crucial for safety assessments in novel food product registrations, as outlined in EU regulations and in countries such as Thailand (European Food Safety Authority n.d.; Notification of the Ministry of Public Health (No. 376) B.E. 2559 2016).

Interestingly, our findings show that 12 weeks of β‐glucan oligosaccharides intake (but not polysaccharides) significantly reduced total cholesterol and LDL levels, with no significant changes in triglyceride or HDL levels. Remarkably, the lipid‐lowering effect persisted for 2 weeks after discontinuing β‐glucan intake. These results suggest that oligosaccharides may offer better efficacy, warranting further investigation. This contrasts with the findings of Bae et al., who reported no effect of β‐glucan molecular weight on serum lipid profiles (Bae et al. 2009). It is essential to note that their study utilized oat β‐1,3/1,4‐glucan oligosaccharides produced through hydrolysis, whereas our study employed fungal β‐1,3/1,6‐glucan produced by gamma irradiation. The cholesterol‐lowering effects of β‐glucans are well‐established in numerous studies (Tiwari and Cummins 2011). Mechanistically, β‐glucans are believed to bind bile acids or increase intestinal viscosity, reducing bile acid reabsorption and enhancing fecal bile acid excretion (Sima et al. 2018; Ellegård and Andersson 2007). However, these effects depend on the degree of viscosity and fiber fermentation (Singla et al. 2024). Khan et al. (2016) demonstrated that gamma irradiation can reduce viscosity while enhancing the bile acid/fat binding capacity of yeast β‐1,3/1,6‐glucan. In contrast, Bae et al. found no linear relationship between molecular weight and the in vitro bile acid/fat binding capacity of oat β‐1,3/1,4‐glucan produced by hydrolysis (Bae et al. 2009). Together, these findings suggest that the structural differences and production methods of β‐glucans may influence their lipid‐lowering effects.

β‐glucans are soluble fibers known to affect bowel movements and defecation (Singla et al. 2024). Previous clinical trials involving both oat and fungal β‐glucans have reported mild flatulence and diarrhea in healthy adults without gastrointestinal disease following β‐glucan consumption (Morales et al. 2021; Wolever et al. 2021). However, our study is the first to demonstrate differential effects on gastrointestinal symptoms between β‐glucan oligosaccharides and polysaccharides. Based on our findings, β‐1,3/1,6‐glucan oligosaccharides produced by gamma irradiation were associated with fewer adverse events compared to β‐glucan polysaccharides. In contrast, a study by Hakkola et al. found no association between perceived gut well‐being and the molecular weight of oat β‐1,3/1,4‐glucan (Hakkola et al. 2021). The high, medium, and low molecular weight (MW) β‐glucans in their study were 1000, 524, and 82 kDa, respectively (Hakkola et al. 2021), while our study used fungal β‐1,3/1,6‐glucan polysaccharides with MWs of around 900 kDa and oligosaccharides with MWs of 5–8 kDa. These differences in β‐glucan structure and molecular weight may explain the contrasting results between our study and theirs.

This study has several strengths. First, the randomized, double‐blind, placebo‐controlled design minimizes bias. Second, the 2000 mg/day dose was determined based on our previous two‐week dose‐escalation study (Muangpracha et al. 2025), which helped mitigate risk to participants. Third, multiple follow‐up assessments (at 4, 8, and 12 weeks of intervention, and 2 weeks after cessation) allowed for careful monitoring of safety and ensured good participant compliance. However, there are some limitations. One challenge was the difficulty in avoiding menstrual periods on follow‐up dates, which led to a few participants having slightly cloudy urine and the presence of RBCs, WBCs, and squamous epithelial cells on the appointment day. Another limitation is that the placebo was an empty capsule, which some participants, particularly those experienced with capsule intake, may have found lighter than regular supplement capsules. Future studies should consider using a placebo capsule filled with starch or maltodextrin to address this issue.

## 5 Conclusions

This study demonstrates that daily supplementation with 2000 mg of β‐glucan oligosaccharides or polysaccharides for 12 weeks is safe for healthy volunteers. Compared to polysaccharides, β‐glucan oligosaccharides are associated with fewer adverse effects and better lipid‐lowering efficacy. Future clinical trials evaluating the efficacy of β‐glucan oligosaccharides and polysaccharides products can safely use doses up to 2000 mg per day.

## Author Contributions

**Numphung Rungraung:** conceptualization (lead), formal analysis (supporting), investigation (lead), writing – original draft (lead). **Niramol Muangpracha:** conceptualization (supporting), formal analysis (supporting), investigation (supporting). **Pakkapong Phucharoenrak:** conceptualization (supporting), formal analysis (supporting), investigation (supporting). **Wai Prathumpai:** conceptualization (supporting), writing – review and editing (supporting). **Dunyaporn Trachootham:** conceptualization (lead), formal analysis (lead), funding acquisition (lead), investigation (equal), methodology (lead), supervision (lead), writing – review and editing (lead).

## Ethics Statement

The study protocol (MU‐CIRB 2023/198.1906) was approved by the Mahidol University Central Institutional Review Board (MU‐CIRB), approval number COA No. MU‐CIRB2023/113.2407. This research was conducted in accordance with the International Council for Harmonization of Technical Requirements for Pharmaceuticals for Human Use Good Clinical Practice (ICH‐GCP) guideline and the Declaration of Helsinki. Informed written consent was obtained from all participants before enrollment.

## Conflicts of Interest

D.T. received a research grant and β‐glucans products from Asia Star Trade Co. Ltd., Thailand. The funders were not involved in the study design, data collection, analysis and interpretation, writing of the manuscript, or in the decision to publish the results. No competing financial interests exist for the other authors. The authors have full control of all primary data.

## Supporting information


**Data S1:** fsn371379‐sup‐0001‐DataS1.docx.

## Data Availability

All data collected in this project have been described in this work. Since the participants only gave consent to report the data summary, no individual data can be shared publicly.
