# Supplementary material for: Differential Safety and Lipid Control Efficacy of β‐1,3/1,6‐Glucan Oligosaccharides and Polysaccharides Derived From Ophiocordyceps dipterigena BCC 2073 in Healthy Volunteers
Source: Food Sci Nutr. 2026 Mar 2;14(3):e71379. doi: 10.1002/fsn3.71379 (PMC12953046; doi:10.1002/fsn3.71379)
Supplement: Supplementary file 1 — Data S1: fsn371379‐sup‐0001‐DataS1.docx. [file FSN3-14-e71379-s001.docx]

**Supplemental data**

**Method**

***β-glucan quantification***

**Total Glucan (α-glucan and β-glucan) Analysis**

The analysis of β-glucan content was performed using the β-Glucan Assay Kit (Yeast and Mushroom) (K-YBGL) from Megazyme (Ireland). Glucan in the culture filtrate was precipitated after mycelial filtration using filter paper No. 1 and then harvested in cold ethanol. The glucan was then redissolved in distilled water and dialyzed using a molecular weight cut-off of 20 kDa. Then weigh 90 mg of the sample and place it into a screw-cap glass tube. Add 2 mL of cold sulfuric acid. Close the cap tightly and mix using a vortex mixer. Place the tube in an ice bath for 2 hours, mixing every 10–15 seconds. Then, add 4 mL of water and mix thoroughly. Add 6 mL of water and mix for an extra 10 seconds. Loosen the cap and boil the mixture at approximately 100°C for 5 minutes. Then, close the cap tightly and continue cooking for 2 hours. Allow the sample to cool to room temperature. Add 6 mL of 8.0 M NaOH, then adjust the volume to 100 mL using 200 mM sodium acetate buffer (pH 4.5). Mix thoroughly before taking a 1.5 mL aliquot of the sample and centrifuge at 13,000 rpm for 5 minutes. Transfer 0.1 mL of the supernatant into a centrifuge tube and add 0.1 mL of exo-1,3 β-glucanase plus β-glucosidase solution. Mix thoroughly and incubate at 40°C for 60 minutes. Add 3 mL of GOPOD reagent buffer and incubate at 40°C for 20 minutes before measuring the absorbance at a wavelength of 510 nm.

**α-Glucan Analysis**: Weigh 100 mg of the sample and place it into a screw-cap glass tube. Add 2 mL of 1.7 M NaOH and incubate in an ice bath for 20 minutes. Add 8 mL of 1.2 M sodium acetate buffer (pH 3.8) and mix immediately. Then, add 0.2 mL of the amyloglucosidase plus invertase solution. Mix thoroughly and incubate at 40°C for 60 minutes. Then add NaOH (volume unspecified), and adjust the volume to 100 mL with water. Mix thoroughly before taking a 1.5 mL aliquot and centrifuge at 13,000 rpm for 10 minutes. Transfer 0.1 mL of the clear supernatant into a glass tube, then add 0.1 mL of 200 mM sodium acetate buffer (pH 4.5), followed by 3 mL of GOPOD reagent buffer. Incubate at 40°C for 20 minutes, then measure the absorbance at a wavelength of 510 nm. The concentration is then calculated by comparing the absorbance with that of the standard solution.

The amount of β-glucan is obtained by subtracting the α-glucan content from the total glucan value. The purity of β-glucan is determined by the amount of β-glucan divided by the total weight.


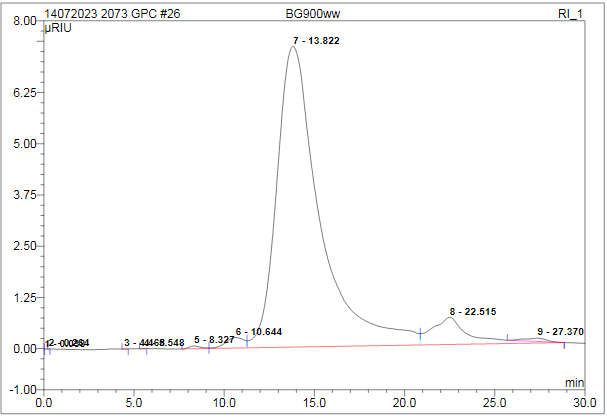

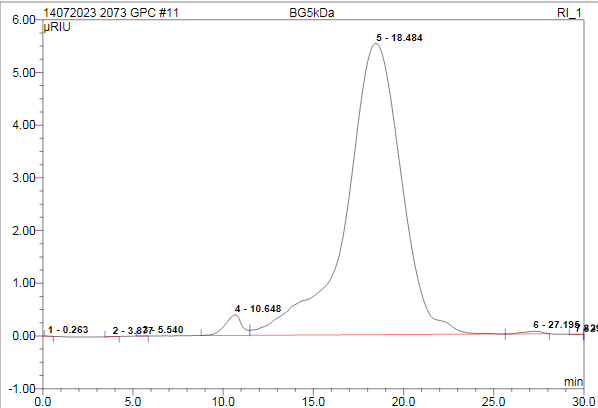


**A B**

**Figure S1** Chromatograms of A: 900 kDa polysaccharide and B: 5 kDa oligosaccharide

using size exclusion chromatography


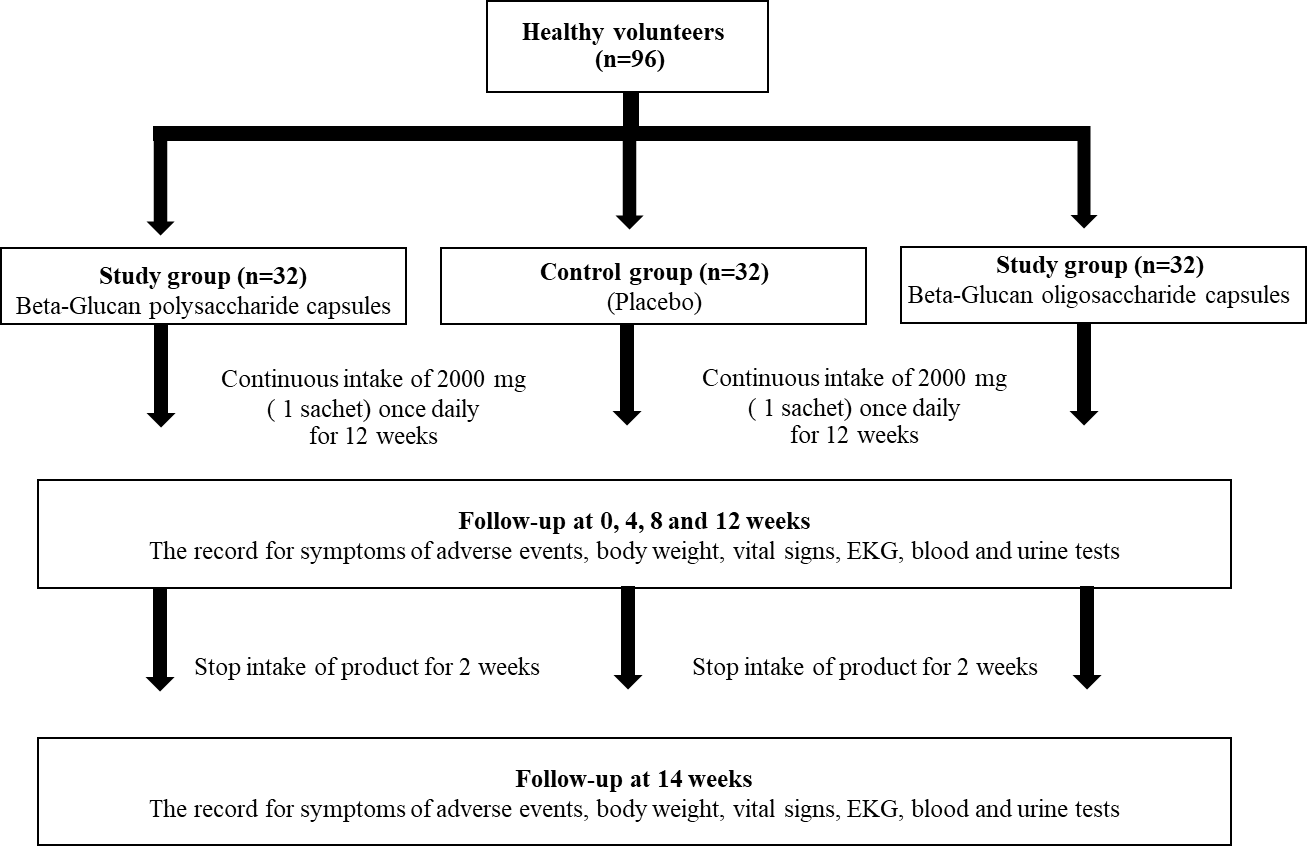


**Figure S2:** Conceptual framework


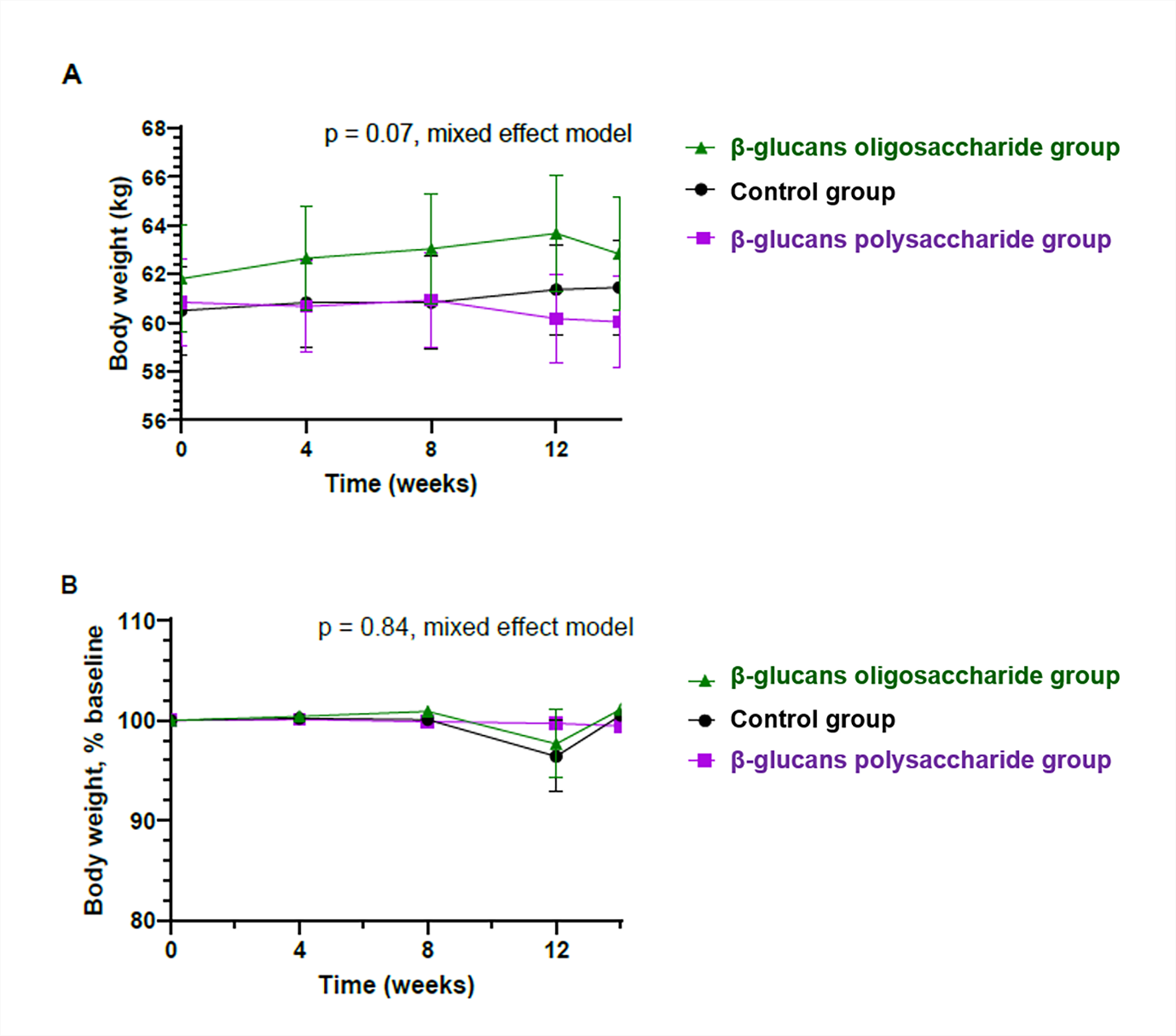


**Figure S3.** Changes in body weight of the volunteers. (**A**) The line graph shows the mean and standard deviation of body weight in the β-glucans oligosaccharide (green), β-glucans polysaccharide (purple), and control (black) groups supplementation for 4, 8, and 12 weeks and 2 weeks after stopping intake (14 weeks), with *p*-value obtained from mixed effect analysis; (**B**) the line graph shows the mean and standard deviation of body weight (% baseline) in the β-glucans oligosaccharide (green), β-glucans polysaccharide (purple), and control (black) groups, with *p*-value obtained from mixed effect analysis.


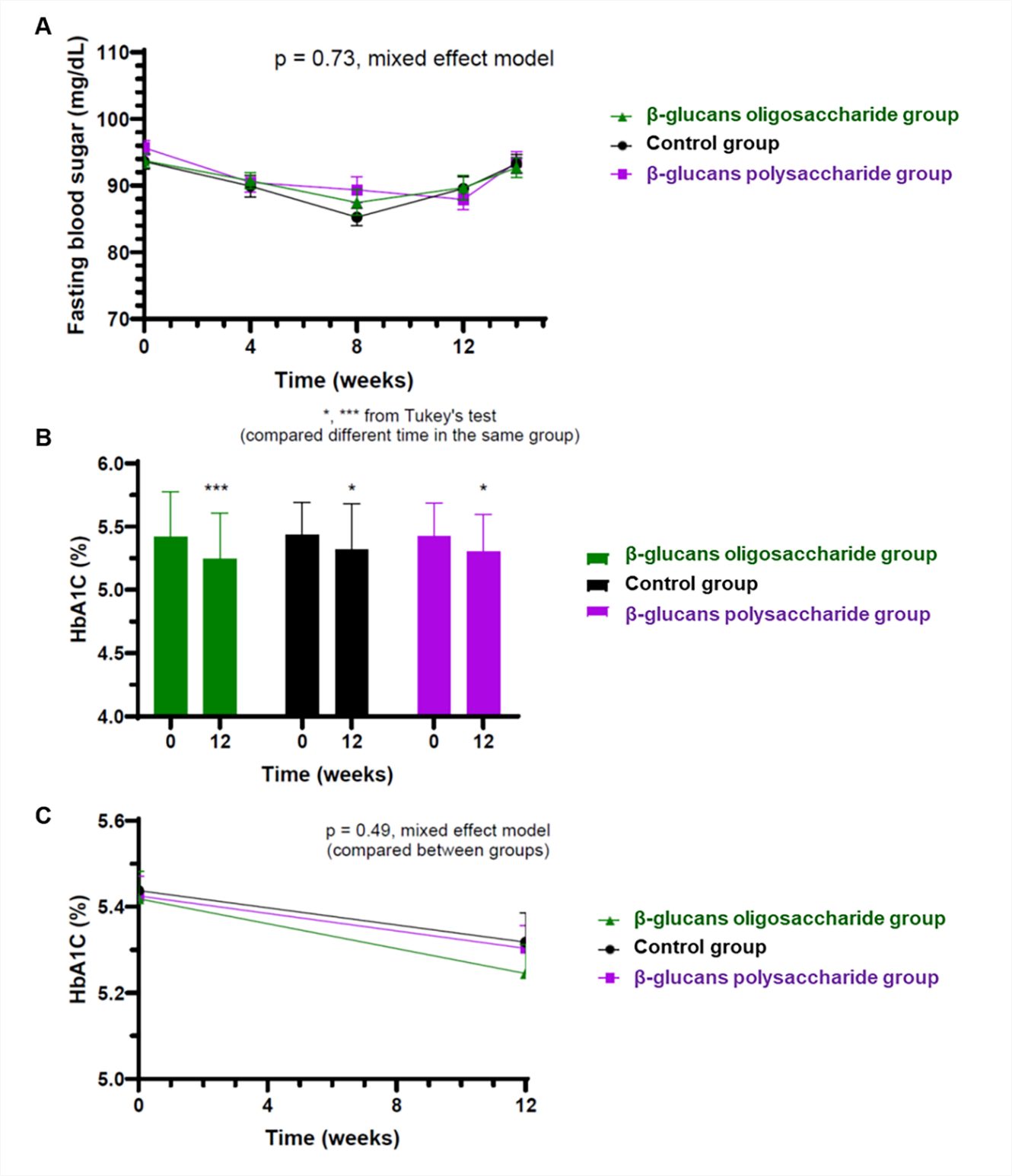


**Figure S4**Changes in fasting plasma glucose and HbA1C after the β-glucans oligosaccharide (green), β-glucans polysaccharide (purple), and control (black) group supplementation (**A**) The line graph shows the mean and standard deviation of fasting plasma glucose at 4, 8, and 12 weeks and 2 weeks after stopping intake (14 weeks), with p-value obtained from mixed effect analysis; (**B**) the bar graph shows the mean and standard deviation of HbA1C at 0 and 12 weeks, p-value analyzed by mixed effect analysis and Tukey’s test (compare different time in the same group); (**C**) The line graph shows the mean and standard deviation of HbA1C at 0 and 12 weeks, with p-value obtained from mixed effect analysis (compared between groups). *, and *** mean p < 0.05 and 0.001, respectively. Abbreviations: HbA1C, glycosylated hemoglobin.


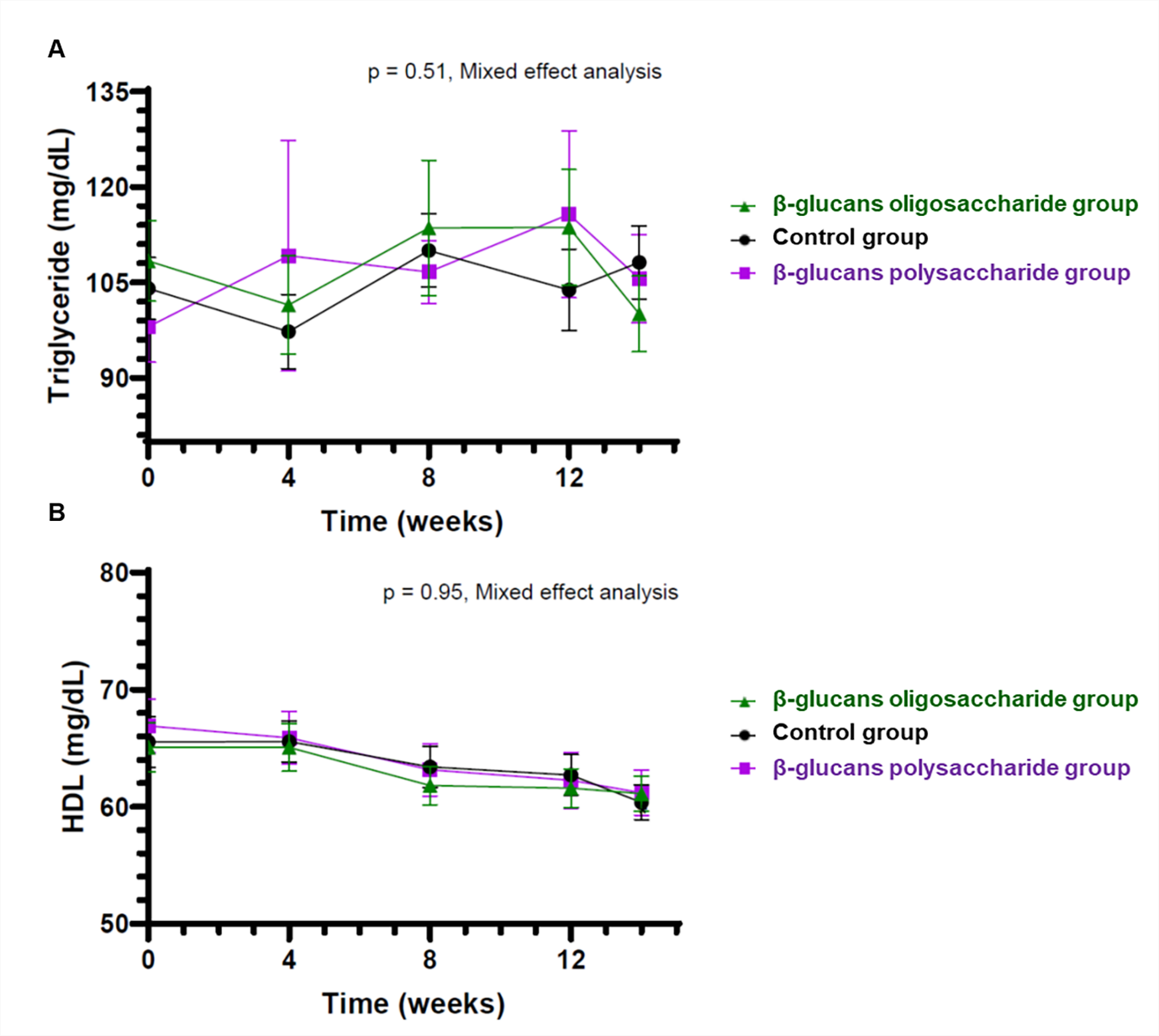


**Figure S5**Changes in triglyceride and HDL levels. The line graph shows the mean and standard deviation of the volunteer’s triglyceride (**A**) and LDL (**B**) after the β-glucans oligosaccharide (green), β-glucans polysaccharide (purple), and control (black) group supplementation for 4, 8, and 12 weeks and 2 weeks after stopping intake (14 weeks), with *p*-value obtained from Mixed effect analysis test. Abbreviations: HDL, high-density lipoprotein.


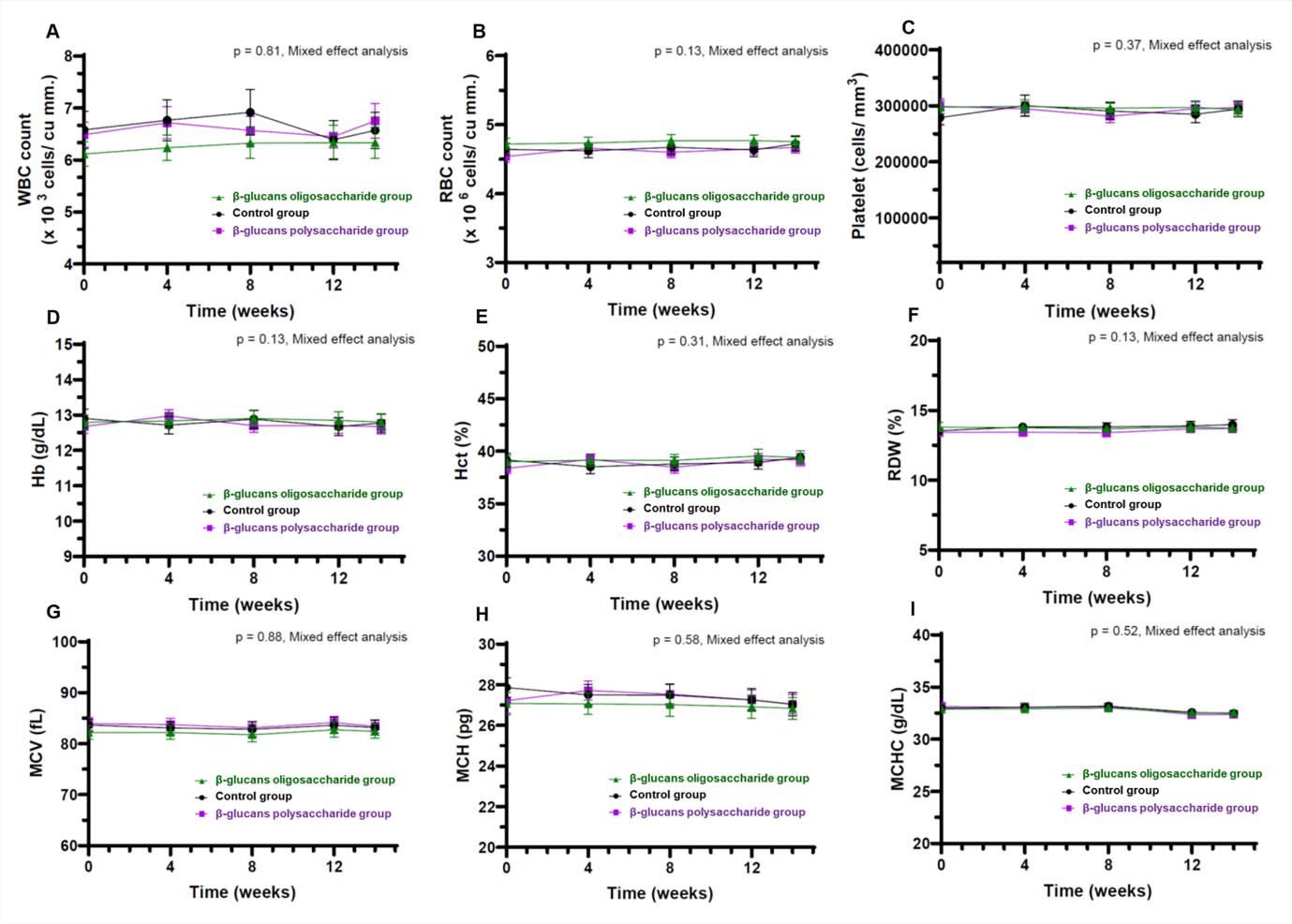


**Figure S6** Changes in hematological parameters. The line graph shows the mean and standard deviation of the volunteer’s WBC (**A**), RBC (**B**), platelet (**C**), Hb (**D**), Hct (**E**), RDW (**F**), MCV (**G**), MCH (**H**), and MCHC (**I**) after the β-glucans oligosaccharide (green), β-glucans polysaccharide (purple), and control (black) group supplementation for 4, 8, and 12 weeks and 2 weeks after stopping intake (14 weeks), with *p*-value obtained from Mixed effect analysis test. Abbreviations: WBC, white blood cell count; RBC, red blood cell count; Hb, hemoglobin; Hct, hematocrit; RDW, red blood cell distribution width; MCV, mean cell volume; MCH, mean cell hemoglobin; MCHC, mean cell hemoglobin concentration.


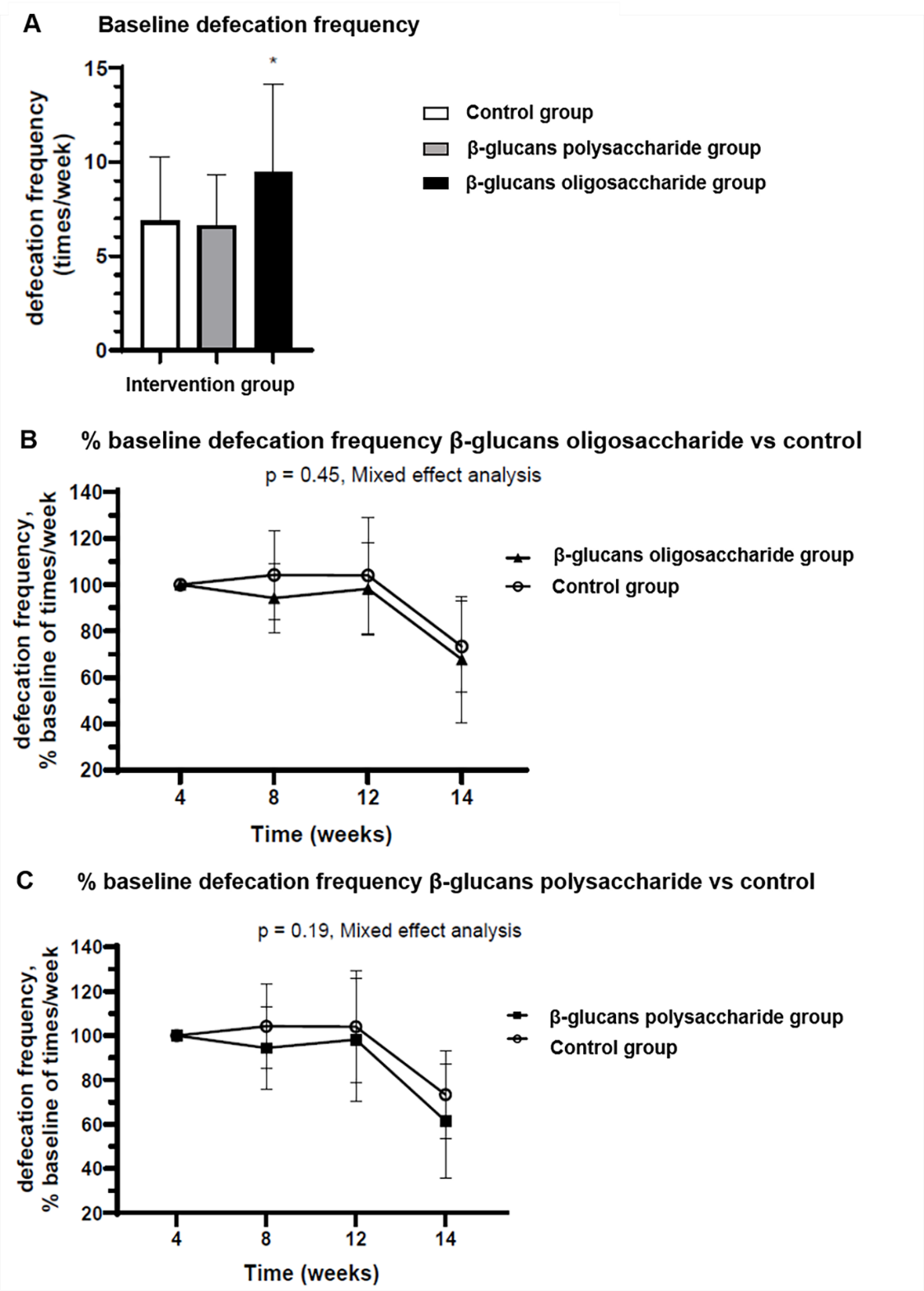


**Figure S7** Changes in defecation frequency based on the subject diary (**A**) The bar graph shows mean and standard deviation of defecation frequency at baseline in control (white), β-glucan oligosaccharides (gray), and β-glucans polysaccharide (black) groups, with * = p < 0.05 from one-way ANOVA and Tukey’s test. (**B**) The line graph shows the mean and standard deviation of the control (), and β-glucans oligosaccharide () group as % baseline, (**C**) The line graph shows mean and standard deviation of the control (), and β-glucans polysaccharide () group as % baseline with p-value from mixed-effect analysis.


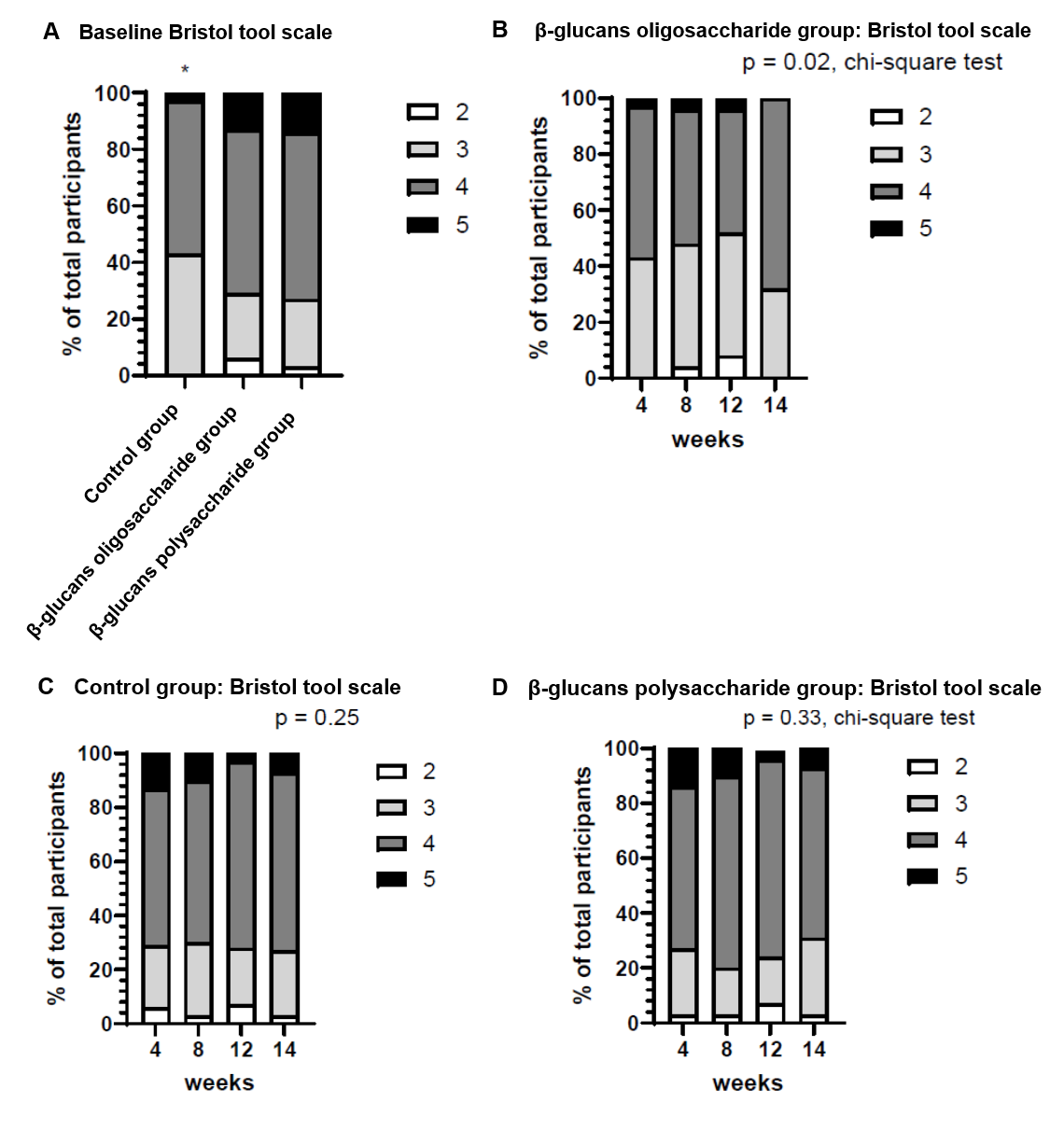


**Figure S8** Changes in stool consistency based on subject diaries The stacked bar shows the percentage of total participants who had Bristol stool scale of 2 (white), 3 (light grey), 4 (dark grey), and 5 (black) at baseline of all groups (**A**), after taking β-glucans oligosaccharide (**B**), placebo (control group) (**C**), and β-glucans polysaccharides (**D**), with *p*-value from chi-square test.


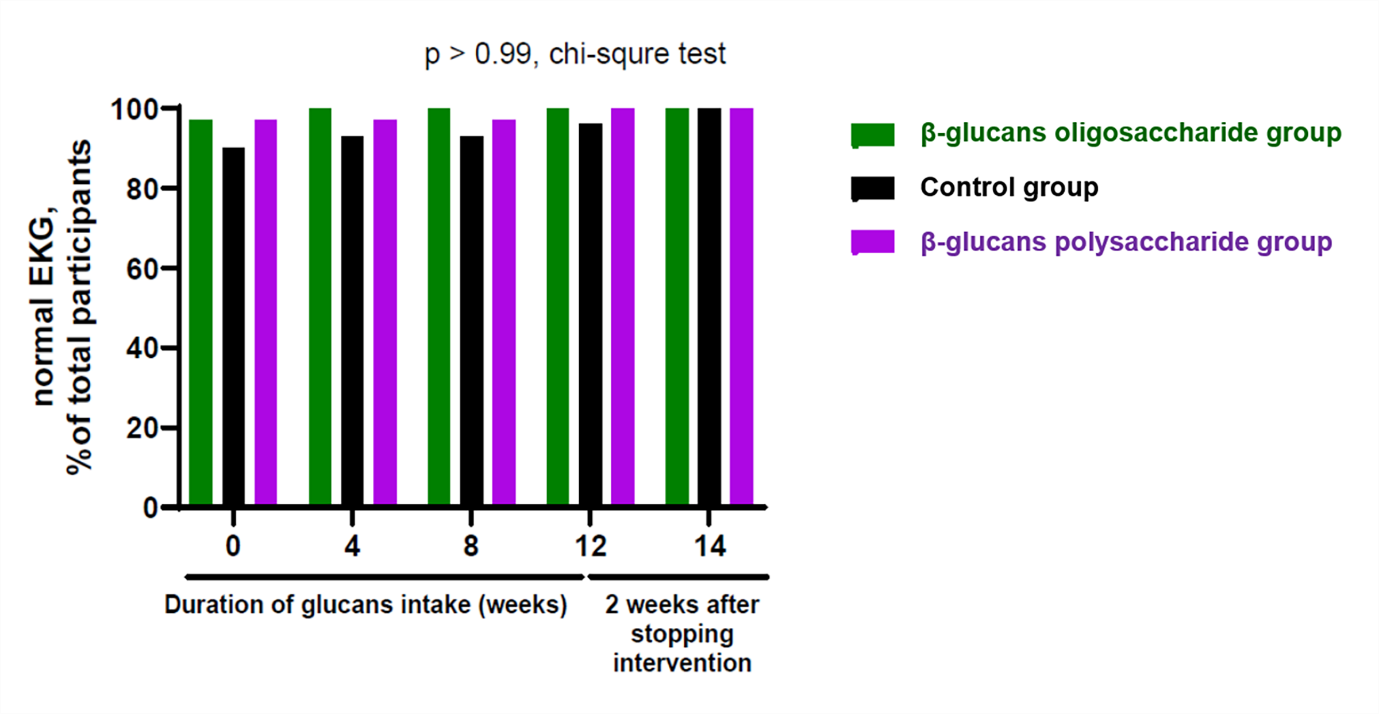


**Figure S9** Changes in EKG. The bar graph shows the percentage of participants with normal EKG characteristics in the β-glucans oligosaccharide (green), β-glucans polysaccharide (purple), and control (black) groups, with *p*-value obtained from the chi-square test.

**Table S1:** Demographic characteristics of the participants at baseline

|  | **β-glucans oligosaccharide Group**  **(n=32)** | **β-glucans polysaccharide Group**  **(n=32)** | **Control**  **Group**  **(n=32)** | **p-value** |
| --- | --- | --- | --- | --- |
| Parameter | N (%) | N (%) | N (%) |  |
| Gender |  |  |  |  |
| Male | 7 (22) | 7 (22) | 7 (22) | >0.99 |
| Female | 25 (78) | 25 (78) | 25 (78) |  |
| No systemic diseases | 32 (100) | 32 (100) | 32 (100) |  |
| No history of smoking | 32 (100) | 32 (100) | 32 (100) |  |
| No history of alcohol drinking | 32 (100) | 32 (100) | 32 (100) |  |
|  | **β-glucans oligosaccharide Group**  **(n=32)** | **β-glucans polysaccharide Group**  **(n=32)** | **Control**  **Group**  **(n=32)** | **p-value** |
| Parameter | Mean ± SD | Mean ± SD | Mean ± SD |  |
| Age (years) | 38.13 ± 8.64 | 37.50 ± 9.41 | 37.53 ± 8.02 | 0.933 |
| BMI (kg/m^2^) | 23.85 ± 4.01 | 23.33 ± 3.17 | 23.33 ± 3.60 | 0.915 |
| Systolic blood pressure (mmHg) | 108.60 ± 10.86 | 111.80 ± 11.31 | 111.10 ± 13.55 | 0.538 |
| Diastolic blood pressure (mmHg) | 75.42 ± 7.76 | 75.90 ± 7.18 | 78.44 ± 9.55 | 0.421 |
| Pulse rate (beats/min) | 74.94 ± 9.54 | 76.83 ± 12.60 | 76.25 ± 11.55 | 0.819 |

Abbreviations: BMI, body mass index; n = the number of participants; p-value from Chi-square test for gender and Kruskal-Wallis tests for other parameters

**Table S2:** Compliance for consumption of capsule supplementation

| **Intervention group** | **Total number of assigned capsules** | **Number of consumed capsules ^1^** | **Adherence based on capsule count**  **(%)** | **Total number of assigned days of consumption** | **Number of consumed days based on self-record ^2^** | **Adherence based on self-record (%)** |
| --- | --- | --- | --- | --- | --- | --- |
| β-glucans oligosaccharide  (n=32) | 336 | 333 ± 7 | 99.10 | 84 | 83 ± 2 | 98.80 |
| β-glucans polysaccharide  (n=32) | 336 | 333 ± 5 | 99.10 | 84 | 83 ± 1 | 98.80 |
| Control  (n=32) | 336 | 333 ± 5 | 99.10 | 84 | 83 ± 1 | 98.80 |

^1^ data obtained by counting the number of capsules in the returned package after completion of the study

^2^ data obtained by self-record of participants in the subject diary

**Table S3** Summary of adverse symptoms.

| **Variables** | **β-glucans oligosaccharide Group**  **(n=32)** | **β-glucans polysaccharide Group**  **(n=32)** | **Control**  **Group**  **(n=32)** |
| --- | --- | --- | --- |
| Subjects with any AEs | 0 | 0 | 0 |
| Specific with AEs |  |  |  |
| Nausea | 0 | 0 | 0 |
| Vomiting | 0 | 0 | 0 |
| Abdominal pain | 0 | 0 | 0 |
| Abdominal discomfort | 0 | 0 | 0 |
| Gas in the GI tract | 0 | 0 | 0 |
| Headache | 0 | 0 | 0 |

Data are shown as the number of subjects with adverse events

**Table S4** Daily food consumption of participants

| **Dietary intake** | **β-glucans oligosaccharide group (n=32)** | | | **β-glucans polysaccharide group (n=32)** | | | **Control group (n=32)** | | | ***p*-value Compared between groups** |
| --- | --- | --- | --- | --- | --- | --- | --- | --- | --- | --- |
|  | **week 4** | **week 12** | **p-value** | **week 4** | **week 12** | **p-value** | **week 4** | **week 12** | **p-value** |  |
| Energy (kcal) | 1142.11 ± 374.13 | 1195.4 ± 456.07 | 0.95 | 1204.79 ± 506.6 | 950.07 ± 334.23 | 0.08 | 1044.82 ± 506.7 | 1141.31 ± 439.96 | 0.75 | 0.69 |
| CHO (g) | 144.22 ± 43.74 | 161.85 ± 77.47 | 0.62 | 140.71 ± 61.68 | 115.98 ± 53.83 | 0.33 | 120.07 ± 75.22 | 120.98 ± 43.15 | >0.9999 | 0.17 |
| SUGAR (g) | 38.29 ± 21.03 | 43.37 ± 38.41 | 0.86 | 37.86 ± 26.62 | 32.10 ± 26.47 | 0.81 | 26.12 ± 19.81 | 29.65 ± 22.94 | 0.95 | 0.16 |
| PRO (g) | 49.18 ± 24.18 | 47.24 ± 12.34 | 0.99 | 58.72 ± 30.49 | 43.20 ± 14.3 | 0.11 | 49.27 ± 24.94 | 60.02 ± 31.7 | 0.37 | 0.53 |
| FAT (g) | 40.94 ± 19.78 | 39.89 ± 18.63 | 0.99 | 45.23 ±  21.68 | 34.82 ± 16.34 | 0.30 | 40.83±  22.96 | 46.37 ±  24.41 | 0.78 | 0.71 |
| CHOLE (mg) | 202.83 ± 133.16 | 247.61 ± 203.98 | 0.83 | 250.69 ±  207.29 | 252.25 ± 180.23 | 0.99 | 280.53 ±  168.25 | 358.44 ±  203.24 | 0.47 | 0.65 |
| FBC (g) | 0.17 ± 0.22 | 0.27 ± 0.29 | 0.68 | 0.33 ±0.55 | 0.16 ± 0.19 | 0.19 | 0.11 ± 0.09 | 0.18 ± 0.23 | 0.86 | 0.28 |
| FBD (g) | 8.59 ± 7.08 | 5.73 ± 2.51 | 0.38 | 7.92 ±7.05 | 4.11 ± 2.3 | 0.13 | 6.13 ± 7.77 | 4.71 ± 2.79 | 0.60 | 0.45 |

Data are shown as mean ± SD. Abbreviations: CHO, carbohydrate; PRO, protein; CHOLE, cholesterol; FBC, crude fiber; FBD, dietary fiber; p-values were obtained from unpaired t-test.
